# Low birth weight as a predictor of adverse health outcomes during adulthood in twins: a systematic review and meta-analysis

**DOI:** 10.1186/s13643-021-01730-5

**Published:** 2021-06-24

**Authors:** Sapha Hassan, Shayesteh Jahanfar, Joseph Inungu, Jeffrey M. Craig

**Affiliations:** 1 Central Michigan University, Mount Pleasant, USA; 2 Department of Public Health and Community Medicine, Tufts School of Medicine, 145 Harrison Ave, Boston, MA 02111 USA; 3 Deakin University, IMPACT – the Institute for Mental and Physical Health and Clinical Translation, School of Medicine, Geelong, Australia

## Abstract

**Background:**

Low birth weight might affect adverse health outcomes during a lifetime. Our study analyzes the association between low birth weight and negative health outcomes during adulthood in twin populations.

**Methods:**

Searches were conducted using databases inclusive of MEDLINE, CINAHL, Web of Science, and EBSCO. Observational studies on twins with low birth weight and adverse health outcomes during adulthood were included. Two reviewers independently screened the papers, and a third reviewer resolved the conflicts between the two reviewers. Following abstract and title screening, full-texts were screened to obtain eligibility. Eligible full-text articles were then assessed for quality using a modified Downs and Black checklist. Studies with a score within one standard deviation of the mean were included in the analysis. A fixed-effect model was used for analysis.

**Results:**

3987 studies were screened describing low birth weight as a risk factor for adverse health outcomes during adulthood for all twelve-body systems (circulatory, digestive, endocrine, lymphatic, muscular, nervous, reproductive, respiratory, skeletal, urinary, and integumentary systems). One hundred fourteen articles made it through full-text screening, and 14 of those articles were assessed for quality. Five papers were selected to perform two meta-analyses for two outcomes: asthma and cerebral palsy. For asthma, the meta-analyses of three studies suggested a higher odds of low birth weight twins developing asthma (OR 1.33, 95% CI 1.24-1.44, I^2^ = 77%). Meta-analysis for cerebral palsy included two studies and suggested a 4.88 times higher odds of low birth weight twins developing cerebral palsy compared to normal birth weight twins (OR 4.88, 95% CI 2.34-10.19, I^2^ = 79%). We could not find enough studies for other adverse health outcomes to pool data for a Forest plot.

**Conclusions:**

The odds of low birth weight were found to be high in both asthma and cerebral palsy. There are not enough studies of similar nature (study types, similar body systems) to ensure a meaningful meta-analysis. We recommend that future research considers following up on twins to obtain data about adverse health outcomes during their adult lives.

## Background

According to the World Health Organization, it is estimated that 15-20% of all births worldwide are low birth weight, which equates to more than 20 million births per year [1]. Low birth weight, defined as being born less than or equal to 2500 grams, is extensively related to a poor child and adult health outcomes [2, 3]. Babies born with more severe low birth weights are more likely to develop chronic illness and difficulty with cognition [3, 4]. One study suggested that lower birth weight is associated with short telomere length and lower cognitive ability [5]. Another study demonstrates that low birth weight is associated with later development of non-insulin-dependent diabetes mellitus, six indicating a positive relationship between being born low birth weight and adverse health outcomes.

Previous studies have confirmed that twin gestations are associated with higher perinatal morbidity and mortality when compared to gestations of singleton pregnancies [6, 7], and multiple pregnancies are among the significant risk factors for preterm births [8]. Babies born in multiple pregnancies are more likely to be low birth weight when compared to their singleton counterparts [9, 10]. In 2015, in the USA, according to the Centers for Disease Control and Prevention (CDC), about 55% of twins were born low birth weight, and about 6% of singletons were reported as low birth weight, utilizing the same threshold for low birth weight singletons and in twins [10]. Thus, twins are at a higher risk of being born low birth weight when compared to singletons, and by extension, are more likely to develop adverse health outcomes that are associated with being born low birth weight [11].

Twin studies give us information about the potential epigenetic etiologies of disease [12–14]. One study examined the association between birth weight and rheumatoid arthritis (RA) in twins discordant for RA, concluding that birth weight was not associated with the development of RA in adult life when adjusted for appropriate confounders [15]. Another study found a positive association between low birth weight and development of type 2 diabetes when comparing within-pair differences in twins, suggesting that genetic factors contribute to outcomes later on in life [16]. Thus, twin studies provide us with valuable information regarding the etiology and epigenetic roots of health and disease.

From the preceding section, it is proven that twins are at higher risk of being born low birth weight and, by extension, are at a higher risk of developing diseases and conditions that are associated with their birth weight. To our knowledge, there is no systematic review that studies the impact of low birth weight on future adult health outcomes in twin populations. Additionally, literature examining these outcomes focuses on one sole outcome rather than grouping these outcomes into a similar category, such as bodily systems. We compared longitudinal outcomes of twins of low birth weight compared with normal birth weight twins. The methodology that our review follows is the Cochrane method of screening, analysis, and quality assessment. To our knowledge, there is no systematic review that uses this method and analyzes how low birth weight affects longitudinal outcomes in twins through the body systems we are investigating; thus, our study is unique in that sense. We studied this association across all body systems: circulatory, digestive, endocrine, lymphatic, muscular, nervous, reproductive, respiratory, skeletal, urinary, and integumentary systems.

### Study question and objective

Our study question attempted to answer this question: Does low birth weight among twins have a long-term impact during adulthood? Our study investigates the association between low birth weight among twins and long-term outcomes during adulthood.

## Methods

The target population of this systematic review is adult twin populations that have data on birth weights. The exposure is low birth weight, and the comparator is average birth weight. The study outcomes are any adult health outcomes classified by the bodily system through clinical measurements. Finally, the study design is further described below.

The methodology follows the MOOSE statement and is explained under seven categories: search strategy, inclusion and exclusion criteria, databases, study selection, data extraction, quality assessment, and statistical analysis.

### Search strategy

Searches were conducted using databases inclusive of MEDLINE, CINAHL, Web of Science, and EBSCO. A comprehensive list of Mesh terms was obtained by three means (see Appendix 2). Firstly, the definition of each concept was extracted from MEDLINE. Secondly, gray literature, conference proceedings, and reference lists of published articles were explored. Thirdly, specialists in the field were consulted to identify the Mesh terms. These included: “low birth weight,” “twin studies,” “longitudinal outcome(s),” adult outcome(s),” “multiple pregnancies,” “twin pregnancy,” and “observational studies.” Boolean logic was used to combine the concepts and eliminate irrelevant articles. Filters were employed to limit the search to observational studies only. The search was limited to English literature.

### Inclusion and exclusion criteria

The target population of this systematic review is adult twin populations (P) that have data on birth weights. The exposure is the low birth weight (I), and the comparator is a normal birth weight (C). The study outcomes are any adult adverse health outcomes, including those related to twelve-body systems (circulatory, digestive, endocrine, lymphatic, muscular, nervous, reproductive, respiratory, skeletal, urinary, and integumentary systems). (O) The bodily system can classify that through clinical measurements. Finally, the study design is observational studies (S). Low birth weight was defined as birth weight less than 2500 grams. Clinical diagnosis of chronic conditions and illnesses by a medical doctor, presented by the authors, was considered to substantiate such conditions.

### Databases

The following databases were searched, from the earliest available date (mentioned in brackets) to March 10, 2018: MEDLINE (1996), Cochrane Central Register of Controlled Trials (1991), CINAHL (1982), Database of abstracts of reviews of effects (1991), Web of Science (1990), and EBSCO (1946). The references of the full papers included in the study analysis were checked to find additional articles. Articles were stored in a reference manager, EndNote, version 18. References were then imported into a systematic review manager, Covidence, and duplicates were deleted.

### Study selection

The screening process was done on Covidence. Two reviewers were involved in the screening process. Two individuals completed the screening; if consensus was not reached about whether to include a study in the subsequent screening or final analysis, a third reviewer determined if a study was included. Cohen’s kappa coefficient for the level of agreement for the first 100 abstracts was 0.78. Differences of opinions were resolved by consensus.

Articles were initially stored in Endnote®, a reference manager, and duplicates were deleted. The remaining articles were transferred to Covidence, an online application designed to facilitate literature screening and data extraction. Two reviewers screened article titles for appropriateness and relevance, with those not meeting the study criteria or recognized as duplicates were eliminated (stage 1a). Abstracts of all potentially relevant articles were then retrieved and screened in a similar manner (stage 1b).

### Data extraction

Articles were excluded or promoted to the next stage based on the responses entered to each question on the relevant form. Data extraction for systematic reviews and original studies involved using a standard data extraction form embedded in Covidence. This form included the following information: Study title, authors, year of publication, country of origin, sample size, enrollment period, outcome reported, maternal age, gestational age, zygosity, and sex of the twins in each group. The outcome reported included the following information: The OR, 95% confidence interval (95% CI), test statistics for the interaction, and statistical significance of the analysis were extracted from selected studies. For papers, not reporting OR and 95% CI, the raw data (i.e., number of events and the total number of samples in the exposed and unexposed groups) were used to estimate OR and 95% CI. For eligible studies, two review authors extracted the data using Covidence. Data were entered into Review Manager Software (RevMan, 2018).

### Quality assessment

The risk of bias and quality assessment of selected studies was assessed through a modified Downs and Black checklist for methodological quality assessment of health care interventions [17]. We chose to utilize this checklist for quality assessment because it was developed specifically for health care interventions. Additionally, this checklist provides an overall quality index and four sub-scales of quality assessment (reporting, external quality, internal validity-bias, and internal validity-confounding). Appendix 1 shows the full checklist that was used in the quality assessment. We did not exclude any study based on quality. Both SJ and SH screened the articles, and JI was the third reviewer. JC was the advisor.

### Statistical analysis

Meta-analyses were conducted using the generic inverse variance with a fixed- or random-effect model in Revman 5.2. We compared the odds between fixed effect and random effect to adjust for weighting. If the effect measure was found to be the same, we reported the fixed effect. The I^2^ test quantified the heterogeneity across individual studies. Low, moderate, or high degrees of heterogeneity were approximated by I^2^ values of 25%, 50%, and 75%, respectively. Reasons for heterogeneity were investigated by eyeballing extreme OR and sensitivity analysis.

We planned to conduct subgroup analyses using the following variables: zygosity (monozygotic or dizygotic), gestational age (yes or no), sex of twins (discordant or concordant), maternal age (yes or no), and twin data comparisons (if low birth weight was compared with singleton or twin standards of birth weight). However, we were unable to do this because of a lack of studies.

Sensitivity analysis was conducted by excluding data points with extreme OR or maximum weight to examine their impact on the overall OR. We were planning to investigate the publication bias using inverted funnel plots. However, the plots can only be drawn if meta-analyses include more than ten studies.

## Results

**Fig. 1:**
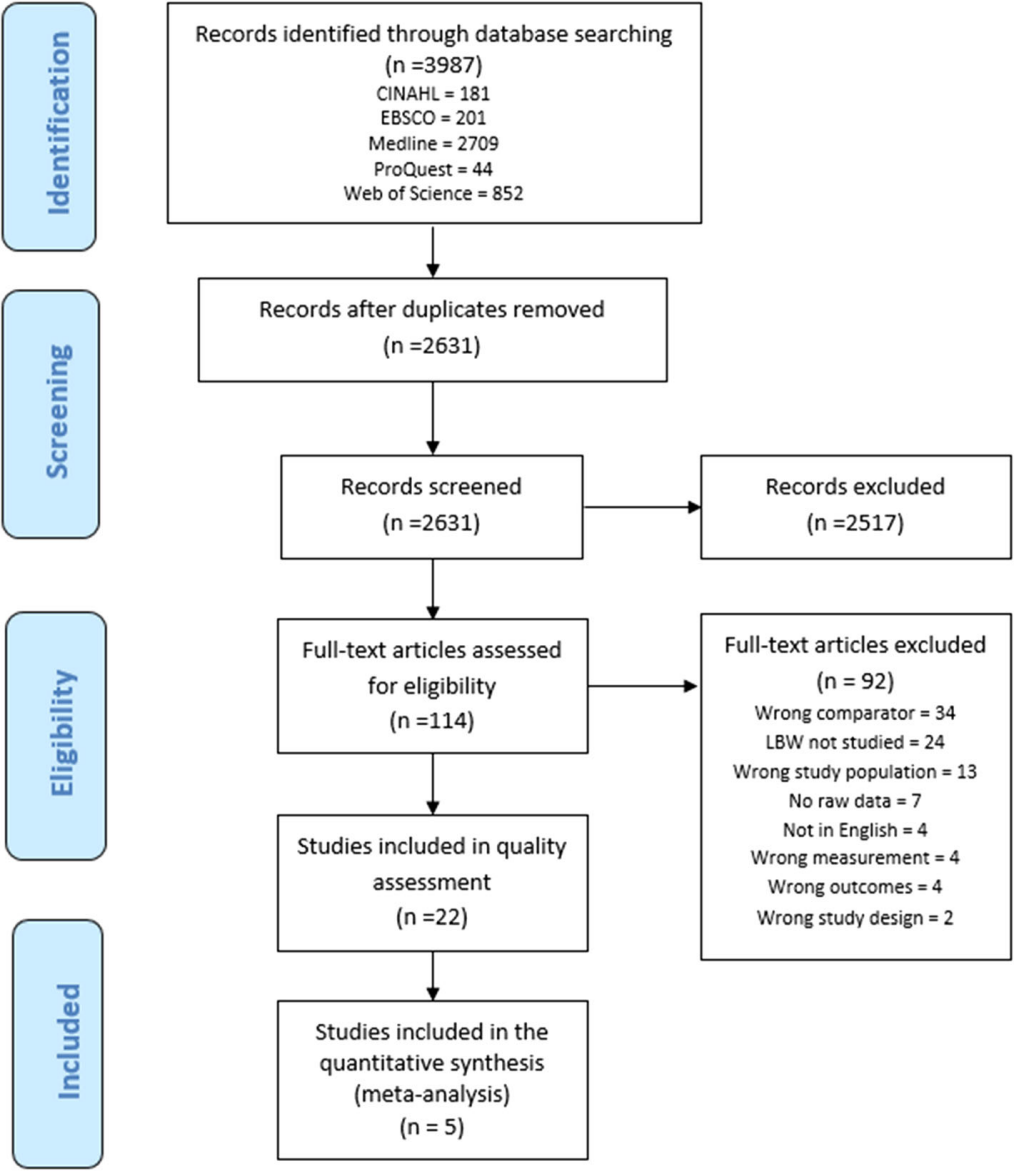
PRISMA flow diagram

Three thousand nine hundred eighty-seven studies were identified through database searching. Duplicates were removed (*n* = 1356), and 2631 abstracts were screened. One hundred fourteen full-text articles were assessed for eligibility, and 22 studies were evaluated for quality using a modified Downs and Black checklist [17] (see Appendix 1 for modified checklist). Studies were included in the quantitative analysis if their scores were within one standard deviation of the mean quality score. Figure 1 shows the PRISMA flow chart summarizing how articles were screened and how many were ultimately eligible for inclusion in this study; Table 1 shows the 14 studies that were eligible for quality assessment. Table 2 presents a description of included studies.

**Table 1:** Quality assessment of the articles reviewed

Study ID (author, year)	Clarity	External validity	Internal validity		Total score
			Bias	Confounding	
Alin Akerman 1995 [18]	4	2	5	2	13
Bergvall 2007 [19]	5	2	5	3	15
Cnattingius 2009 [20]	5	2	5	3	15
De Zeeuw 2012 [21]	4	2	5	2	13
Groen-Blokhuis 2011 [22]	3	2	5	2	12
Hestbaek 2003 [23]	4	2	5	2	13
Hultman 2007 [24]	4	2	5	3	14
Gardner 1995 [25]	3	2	5	2	12
Ortqvist 2009 [11]	5	2	5	3	15
Rasanen 2000 [26]	5	2	5	3	15
Suvanand 1997 [27]	3	2	5	3	15
Villamor 2009 [28]	5	2	5	3	15
Williams 1996 [29]	4	2	5	3	14
Yokoyama 2007 [30]	5	2	4	3	14
**Median (range)**	4 (3-5)	2 (2-2)	5 (4-5)	3 (2-3)	13.5 (12-15)

**Table 2:** Description of the articles included in meta-analyses. All study designs are cross-sectional, and all studies used singleton standards to classify low birth weight

Study ID (author, year)	Country	Number of participants	Enrollment period	Outcome	Maternal age	Gestational age	Zygosity (MZ/DZ)	Sex (MM/MF/FF)
Ortqvist 2009 [11]	Sweden	10918	2004-2007	Asthma	≤ 19: 18.4% 20-24: 16.1% 25-29: 13.9% 30-34: 13.9% ≥ 35: 11.8%	≤31: 24.8% 32-34: 18.1% 35-36: 14.1% 37-38: 11.7% 39-40: 10.7% ≥ 41: 12.9%	MZ: 13.4% DZ: 28.4%: Unknown: 11.7%	-
Rasanen 2000 [26]	Finland	4502	1975-1979	Asthma	< 25: 31.4% 25-30: 38.8% > 30: 29.8%	< 33: 7.1% 33-36: 29.0% 37-40: 60.6% > 40: 3.3%	-	-
Villamor 2009 [28]	Sweden	32580	1926-1958	Asthma	< 20: 2.7% 20-24: 17.0% 25-29: 29.9% 30-34: 27.4% ≥ 35: 22.9% Missing: 0.1%	31-34: 12.9% 35-36: 20.0% 37-41: 58.7% 42-45: 3.5% Missing: 4.8%	-	MM: 47.2% FF: 52.8%
Gardner 1995 [25]	USA	1079	1959-1966	Cerebral palsy	-	-	MZ: 31.9% DZ: 58.6% Unknown: 9.4%	Concordant: 63.9% Discordant: 36.1%
Suvanand 1997 [27]	India	250	1993-1994	Cerebral palsy	-	-	-	-

Although 14 studies were eligible for inclusion in the final model, the outcomes that these studies looked into were distinct from each other; outcomes included attention-deficit disorders, colorectal cancer, and motor development. Each of these papers suggested a positive correlation between low birth weight in twins and the outcomes above. In order to be included in the meta-analyses, we needed at least two studies per body system to study the impact of low birth weight on longitudinal outcomes that are a part of each body system. Thus, two outcomes that are a part of two different body systems were included in the final meta-analysis: asthma (respiratory system) and cerebral palsy (nervous system). Tables 2 and 3 include descriptions of the last five articles that were included in the meta-analyses. The five articles included were all cross-sectional studies using data from four countries, consisting of over 35,000 twin participants.

**Table 3:** Impact of low birth weight on longitudinal outcomes by body system

Body system	Outcome	Number of studies	Number of participants	OR (95%CI)	Heterogeneity (%)
**Nervous**	Cerebral palsy	2	1318	4.88 (2.34-10.19)	79%
**Respiratory**	Asthma	3	37,008	1.33 (1.24-1.44)	77%

### Asthma

**Fig. 2:**
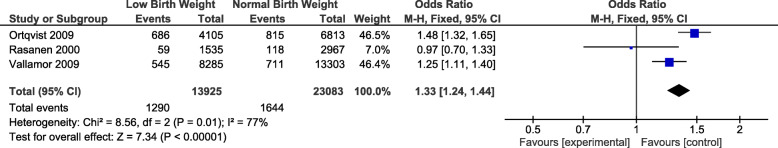
Meta-analysis for association between low birth weight and development of asthma

Asthma is defined as a respiratory condition marked by spasms in the bronchi of the lungs, causing difficulty in breathing. It usually results from an allergic reaction or other forms of hypersensitivity. Figure 2 shows the meta-analysis for the association between low birth weight and the development of asthma later on in life. Three studies were included in this meta-analysis, with just over 37,000 twins being studied. A fixed-effect model was used, and the meta-analysis showed I^2^ = 77% and OR (95% CI) = 1.33 (1.24-1.44). A random-effect model was tested to adjust weighting. However, no change in the effect was observed. Hence, the fixed effect is reported. However, the results were significant (*p* < 0.00001); this outcome shows high heterogeneity and ultimately favored low birth weight.

### Cerebral palsy

**Fig. 3:**
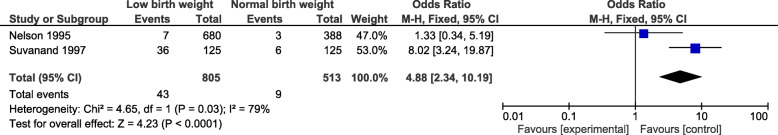
Meta-analysis for the association between of low birth weight and development of cerebral palsy

Table 3 lists the studies used to analyze outcomes of cerebral palsy. Figure 3 shows the meta-analysis for the association between low birth weight in twins and the development of cerebral palsy. This meta-analysis included two studies that looked at about 1300 twin participants. A fixed-effect model was used and yielded I^2^ = 79% and OR (95% CI) = 4.88 (2.34-10.19). A random-effect model was tested to adjust the weight. However, the effect measure did not change. Thus a fixed-effect model was kept and is shown in Fig. 3.

**Fig. 4:**
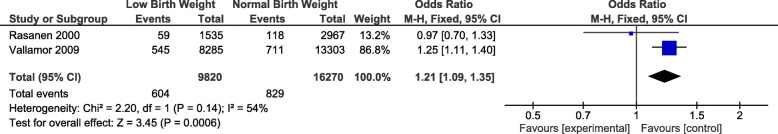
Sensitivity analysis investigating the association between low birth weight and development of asthma. Ortqvist 2009 was not included in this analysis

We performed sensitivity analysis on the asthma outcome due to having more than two studies in our meta-analysis. Sensitivity analysis was impossible for the cerebral palsy outcome due to not eliminating a study, as there were only two studies eligible for the meta-analysis. However, we were able to perform sensitivity analysis for the asthma outcome. Figure 4 shows the meta-analysis without Ortqvist 2009 included. This study provided 46.5% of the weight of the comparison; when eliminating the study, heterogeneity decreased from 77 to 54%, and new OR (95% CI) is 1.21 (1.09-1.35), previously was OR (95% CI) was 1.33 (1.24-1.44).

### Other studies

Table 4 shows all these studies were longitudinal studies from European or Asian countries (e.g., Denmark, Japan). Various outcomes were studied; hence, meta-analysis was not possible—the majority adjusted for zygosity as well as other socio-demographic characteristics.

**Table 4:** Characteristics of studies that were not included in the meta-analysis

Author, year	Study design	Confounders	Outcome	Result
Antoniades 2003 [17]	Cohort study	Height, age	Bone Mineral density (BMD) and bone mineral content (BMC) at the lumbar spine, Hip and forearm	No association was found with LBW
Bergvall 2007 [31]	Cohort study	Zygosity and socioeconomic factors, genetic factors, shared familial environment, BMI	Hypertension	Decreased birth weight was found to be associated with increased risk of hypertension
Cnattingius 2009 [19]	Cohort study	Mother’s short status	Late fetal death rates	The risk of late fetal death is greatly increased in twin fetuses
De Zeeuw 2012 [20]	Cohort study	Mode of delivery, gestational age and zygosity	Educational achievement in primary school	Low birth weight were the most important risk factors for lower educational achievement of twins in primary school
Groen-Blokhuis 2011 [21]	Cohort study	Zygosity	Attention problems	Association of birth weight and attention problems represented a causal relationship
Hestbaek 2003 [22]	Cohort study	Birth factors and anthropometric measures in adolescence	Low back pain	The odds ratio for the lifetime prevalence of low back pain increases from 1.21 (0.94-1.56) for a birth weight of 2000-2500 g to 1.97 (1.35-2.88) for a birth weight of > 3500 g compared to the smallest weight group (< 2000 g) in males, whereas there is a small statistically insignificant, positive association for females.
Hultman 2007 [23]	Cohort study	Fetal growth restriction	Attention-deficit/hyperactivity disorder in childhood and early adolescence	Low birth weight was a risk factor for symptoms of attention-deficit/hyperactivity disorder and the associations did not diminish when we controlled for genetic influence
Williams 1996 [24]	Cohort study	Gestational age	Cerebral palsy	The relative risk was greatest in twins weighing more than 2499 g (4.5). However, after adjusting for reduced birthweight of twins it was the relative risk of twins weighing less than 1400 g that was significantly increased.
Yokoyama 2007 [29]	Cohort study	Gestational age	Motor development in early life	The mean age at achieving milestones was slower in twins with normal birth weight than singletons

## Discussion

Our review investigated published articles that explored the association between low birth weight and adverse health outcomes during adulthood. To our knowledge, there is no systematic review that examined this particular exposure and subsequent outcomes in twins.

Our review indirectly explored the concept of the fetal origins of adult disease (FOAD), initially popularized by Dr. David Barker in the twentieth century [32], and how it applies to twin studies. Dr. Barker initially observed that events during early development and intrauterine environmental exposures impact the risk of development of disease in adulthood. The first evidence of the validity of this hypothesis was a study published in 1989 that showed that low birth weight was associated with an increased risk of coronary artery disease [33]. This hypothesis has been applied to a number of studies to understand if there is a correlation between early developmental factors and adult health outcomes, particularly the development of chronic disease [32]. Twin studies, in particular, are valuable to understanding the FOAD hypothesis as they control for genetic and environmental confounding factors [14, 31], thus helping understand how epigenetic factors apply to health outcomes [12]. Twins also share an intra-uterine environment and can share a childhood environment if not separated at birth.

### Asthma

Because our methodology did not yield many studies, we could only perform two meta-analyses for two outcomes. The first meta-analysis was performed on three twin studies to assess the development of asthma in normal vs. low birth weight twin participants. This meta-analysis (shown in Fig. 2) showed high heterogeneity (I^2^ = 77%) among the three included studies. Thus, there was high variation among these three studies. It is already known that children born with low gestational age or low birth weight are at an increased risk of asthma [34, 35]. Furthermore, it is known that twins are more likely to be born at a lower gestational age and lower birth weight than their singleton counterparts [36]. Although this fact was found valid in several past studies; interestingly, our results show high heterogeneity across the three studies that were assessed in our review.

Asthma is a chronic disease that is typically developed during childhood [29]. The Copenhagen Studies on Asthma in Childhood concluded that 40% of lower lung function cases had asthma present at birth; 26 in other words, children who developed asthma later in life (in this study, at the age of 7) demonstrated lung function deficits in the neonatal period, suggesting support for the hypothesis that later chronic disease can be predicted by early developmental factors [31]. However, other studies have suggested the opposite; these studies essentially attribute asthma development more to environmental exposures rather than to lung function at birth and subsequent lung functioning later on in life [37, 38].

Although this is not an exhaustive review on all studies investigating asthma development, these examples of conflicting results can explain that confounders are to be included to reduce the bias. After following the previously described methodology, we only had three studies in our review, which can also be attributed to high variation in results. Thus, to concretely conclude that there is a positive or negative association between low birth weight and the development of asthma, more studies need to be done to assess this relationship.

### Cerebral palsy

Cerebral palsy is a lifelong physical disability associated with movement and posture disorders and impairments in communication, intellectual ability, and neurological functioning [32]. Worldwide, cerebral palsy is estimated to affect 17 million people, affecting about 1 in 500 neonates [39, 40]. The onset of cerebral palsy is during childhood, and there is currently no cure for the disorder [39, 41]. After following the methodology for our present review, we performed a meta-analysis on two articles that investigated the impact of low birth weight on cerebral palsy development in twin study participants.

Although studies have been conducted for several years regarding the etiology and pathways of cerebral palsy, there is still little known about the risk factors of developing the disorder [40]. However, it is known that multiple pregnancies increase the risk of cerebral palsy twofold in each twin, and twins conceived via in vitro fertilization each have a fourfold risk of developing cerebral palsy, mainly because of the predisposition to cerebral damage that twins have when compared to singletons [42, 43]. In twins, zygosity and sex pairing have previously been studied in cerebral palsy development and survival [44]. This study showed that the prevalence of cerebral palsy was higher in the low birth weight and same-sex twin groups, suggesting evidence for the role of low birth weight in the disorder’s etiology [44].

The high heterogeneity observed in the meta-analysis (shown in Fig. 3) for the association between low birth weight and development of cerebral palsy can be attributed to several factors, primarily due to the lack of studies that assessed the association between low birth weight and cerebral palsy. Past studies have indicated that there is a correlation between the two. Still, after going through our review’s methodology, we could only perform a meta-analysis for two studies for this particular outcome. Thus, this likely affected the variation of results across the two studies. To have more valid and reliable results for this meta-analysis, more studies are necessary.

### Clinical implications and limitations of study

The above results can help adopt preventative measures for the development of asthma. Early intervention to prevent asthma can be done in a more aggressive manner when twins are born low birth weight. Cerebral palsy, however, is largely not preventable; there are few underlying causes of cerebral palsy that have been confirmed through research [45–50]. The present review further suggests that studies on the etiology and possible prevention methods of cerebral palsy should be explored and researched.

The present study included several limitations. Firstly, the number of studies that were included in the final review is low. It can be argued that reliable conclusions cannot be made due to the low number of studies as well as their corresponding heterogeneity. Though we were able to make conclusions for two distinct adult health outcomes, differences in diagnosis were not considered, which is a potential confounder that was not adjusted for in this review. It is possible that diagnosis of cerebral palsy and asthma may vary across countries and clinics. Therefore, this may have affected results.

The quality of evidence in this review was 13.5 ± 1.14 and range of 12 to 15. The maximum total score of short versions of Dawn and Black’s quality assessment criteria is 18. Hence, the quality of studies entered into this review might not be optimal.

## Conclusion

In conclusion, the present systematic review screened almost 4000 articles to study the association between low birth weight and long-term health outcomes in both children and adults. The two outcomes assessed were asthma and cerebral palsy, and we were able to conclude that low birth weight twins were more likely to develop these outcomes when compared to their normal birth weight counterparts. It is important to note that there were a low number of studies that passed inclusion criteria; thus, we recommend that future twin studies collect data regarding low birth weight as a potential risk factor for developing longitudinal outcomes to draw more viable conclusions. According to one of the reviewers of this paper and I quote, “There is a need to connect maternal-child health outcomes with the noncommunicable disease better later in life. It is tempting to think about these categories of conditions as fully separate and unrelated, but as we establish relationships between health in early and later life, we will better understand how to prevent the exploding epidemic of noncommunicable disease in nearly every country in the world.”

## Data Availability

Not applicable

## Appendix 1

### Modified Downs and Black quality assessment tool

**Study clarity:**

1. Is the objective clearly described?
2. Are the main outcomes to be measured clearly described?
3. Is the exposure clearly described?
4. Are the principal confounders in each group of subjects to be compared clearly described?
5. Are the main findings of the study clearly described?

**External validity:**

6) Were the subjects asked to participate in the study representative of the entire population from which they were recruited?
7) Was there an overall participation rate of at least 70%?

**Internal validity – bias:**

8) If any of the results of the study were based on data dredging, was this made clear?
9) In case-control studies, is the time period between the exposure and outcome the same for cases and controls? In cohort studies, do the analyses adjust for different lengths of follow-up for subjects?
10) In case-control studies, was the exposure misclassification likely to bias the reported association toward the null? In cohort studies, did the exposure status change during the follow-up?
11) Were the statistical tests used to assess the main outcomes appropriate?
12) Were the main outcome measurements clearly described, and valid and reliable?

**Internal validity – confounding (selection bias):**

13) In case-control studies, were the cases and controls recruited from the same population? In cohort studies, were study subjects in different exposure groups recruited from the same population?
14) Were study subjects recruited over the same period of time?
15) Was there adequate adjustment for confounding in the analyses from which the main findings were drawn?

## Appendix 2

### Search strategy

1. exp Pregnancy, Multiple/ or exp Twins/
2. exp Birth Weight/
3. ((birth weight or birthweight) adj3 discordan*).mp. [mp=title, abstract, original title, name of substance word, subject heading word, protocol supplementary concept, rare disease supplementary concept, unique identifier]
4. ((birth weight or birthweight) adj3 differen*).mp. [mp=title, abstract, original title, name of substance word, subject heading word, protocol supplementary concept, rare disease supplementary concept, unique identifier]
5. or/2-4
6. 1 and 5
7. exp Epidemiologic studies/
8. (cohortadj (study or studies)).tw.
9. Cohort analy$.tw.
10. (Follow up adj (study or studies)).tw.
11. (observationaladj (study or studies)).tw.
12. Longitudinal.tw.
13. Retrospective.tw.
14. 7-15
15. 6 and 14
16. All bodily systems and similar terminology related to them were added. The search strategy is truncated for consideration related to space.

## Abbreviations

MEDLINE: Medical Literature Analysis and Retrieval System Online
CINAHL: Cumulative Index to Nursing and Allied Health Literature
EBSCO: Elton B. Stephens Company
CDC: Centers for Disease Control and Prevention
RA: Rheumatoid arthritis
MOOSE: Meta-analysis of Observational Studies in Epidemiology
PRISMA: Preferred Reporting Items for Systematic Reviews and Meta-Analyses
FOAD: Fetal origins of adult disease

## References

[1] World Health Organization (2014). Global Nutrition Targets 2025: Low birth weight policy brief.

[2] Tan Q, Frost M, Heijmans BT, von Bornemann Hjelmborg J, Tobi EW, Christensen K, Christiansen L (2014). Epigenetic signature of birth weight discordance in adult twins. BMC Genomics.

[3] Hack M, Flannery DJ, Schluchter M, Cartar L, Borawski E, Klein N (2002). Outcomes in young adulthood for very-low-birth-weight infants. N Engl J Med.

[4] Darlow BA, Horwood LJ, Pere-Bracken HM, Woodward LJ (2013). Psychosocial outcomes of young adults born very low birth weight. Pediatrics..

[5] Strohmaier J, van Dongen J, Willemsen G, Nyholt DR, Zhu G, Codd V, Novakovic B, Hansell N, Wright MJ, Rietschel L, Streit F, Henders AK, Montgomery GW, Samani NJ, Gillespie NA, Hickie IB, Craig JM, Saffery R, Boomsma DI, Rietschel M, Martin NG (2015). Low birth weight in MZ twins discordant for birth weight is associated with shorter telomere length and lower IQ, but not anxiety/depression in later life. Twin Res Hum Genet.

[6] Kilpatrick SJ, Jackson R, Croughan-Minihane MS (1996). Perinatal mortality in twins and singletons matched for gestational age at delivery at ≥30 weeks. Am J Obstet Gynecol.

[7] Jahanfar S, Lim K, Ovideo-Joekes E (2017). Birth weight discordance and adverse perinatal outcomes. J Perinat Med.

[8] Mizrahi M, Furman B, Shoham-Vardi I, Vardi H, Maymon E, Mazor M (1999). Perinatal outcome and peripartum complications in preterm singleton and twins deliveries: a comparative study. Eur J Obstet Gynecol Reprod Biol.

[9] 2015 Annual Report: Low Birth Weight. United Health Foundation. https://www.americashealthrankings.org/explore/2015-annual-report/measure/birthweight/state/ALL. Accessed 20 July 2019.

[10] National Vital Statistics Reports (2016). Centers for Disease Control and Prevention.

[11] Ortqvist AK, Lundholm C, Carlstrom E, Lichtenstein P, Cnattingius S, Almqvist C (2009). Familial factors do not confound the association between birth weight and childhood asthma. Pediatrics..

[12] Bell JT, Saffery R (2012). The value of twins in epigenetic epidemiology. Int J Epidemiol.

[13] Brix TH, Hegedus L (2011). Twins as a tool for evaluating the influence of genetic susceptibility in thyroid autoimmunity. Ann Endocrinol (Paris).

[14] Tan Q, Christiansen L, von Bornemann HJ, Christensen K (2015). Twin methodology in epigenetic studies. J Exp Biol.

[15] Svendsen AJ, Kyvik KO, Houen G, Nielsen C, Holst R, Skytthe A, Junker P (2014). Newborn infant characteristics and risk of future rheumatoid arthritis: a twin-control study. Rheumatol Int.

[16] Johansson S, Iliadou A, Bergvall N (2008). The association between low birth weight and type 2 diabetes - contribution of genetic factors. Epidemiology.

[17] Downs SH, Black N (1998). The feasibility of creating a checklist for the assessment of the methodological quality both of randomised and non-randomised studies of health care interventions. J Epidemiol Community Health.

[18] Alin Akerman B. Eight-year follow-up of cognitive development in 33 twin pairs. Acta Genet Med Gemellol (Roma) [Internet]. 1995;44(3–4):179–88. Available from: http://ovidsp.ovid.com/ovidweb.cgi?T=JS&PAGE=reference&D=med3&NEWS=N&AN=8739729. 8739729

[19] Bergvall N, Iliadou A, Johansson S, de Faire U, Kramer MS, Pawitan Y, Pedersen NL, Lichtenstein P, Cnattingius S (2007). Genetic and shared environmental factors do not confound the association between birth weight and hypertension: a study among Swedish twins. Circulation.

[20] Cnattingius S, Lundberg F, Sandin S, Gronberg H, Iliadou A (2009). Birth characteristics and risk of prostate cancer: the contribution of genetic factors. Cancer Epidemiol Biomark Prev.

[21] de Zeeuw EL, van Beijsterveldt CE, de Geus EJ, Boomsma DI (2012). Twin specific risk factors in primary school achievements. Twin Res Hum Genet.

[22] Groen-Blokhuis MM (2011). Evidence for a causal association of low birth weight and attention problems. J Am Acad Child Adolesc Psychiatry.

[23] Hestbaek L, Leboeuf-Yde C, Kyvik KO, Manniche C (2003). Is low back pain in youth associated with weight at birth? A cohort study of 8000 Danish adolescents. Dan Med Bull.

[24] Hultman CM (2007). Birth weight and attention-deficit/hyperactivity symptoms in childhood and early adolescence: a prospective Swedish twin study. J Am Acad Child Adolesc Psychiatry.

[25] Gardner MO, Goldenberg RL, Cliver SP, Tucker JM, Nelson KG, Copper RL. The origin and outcome of preterm twin pregnancies. [Internet]. Vol. 85, Obstetrics and gynecology. UNITED STATES: Department of Obstetrics and Gynecology, University of Alabama at Birmingham; 1995. p. 553–7. Available from: http://ovidsp.ovid.com/ovidweb.cgi?T=JS&PAGE=reference&D=med3&NEWS=N&AN=7898832. 10.1016/0029-7844(94)00455-M 7898832

[26] Rasanen M, Kaprio J, Laitinen T, Winter T, Koskenvuo M, Laitinen LA. Perinatal risk factors for asthma in Finnish adolescent twins. Thorax. 2000;55(1):25–31. 10.1136/thorax.55.1.25 PMC1745589 10607798

[27] Suvanand S, Kapoor SK, Reddaiah VP, Singh U, Sundaram KR. Risk factors for cerebral palsy. Indian J Pediatr. 1997;64(5):677–85. 10.1007/BF02726124. 10.1007/BF02726124 10771902

[28] Villamor E, Iliadou A, Cnattingius S. Is the association between low birth weight and asthma independent of genetic and shared environmental factors? Am J Epidemiol. 2009;169(11):1337–43. 10.1093/aje/kwp054. Epub 2009 Apr 8. 10.1093/aje/kwp054 19357326

[29] Williams K, Hennessy E, Alberman E (1996). Cerebral palsy: effects of twinning, birthweight, and gestational age. Arch Dis Child Fetal Neonatal Ed.

[30] Yokoyama Y, Wada S, Sugimoto M, Saito M, Matsubara M, Sono J (2007). Comparison of motor development between twins and singletons in Japan: a population-based study. Twin Res Hum Genet.

[31] Antoniades L, MacGregor AJ, Andrew T, Spector TD (2003). Association of birth weight with osteoporosis and osteoarthritis in adult twins. Rheumatology (Oxford).

[32] Barker DJ, Winter PD, Osmond C, Margetts B, Simmonds SJ (1989). Weight in infancy and death from ischaemic heart disease. Lancet..

[33] Calkins K, Devaskar SU (2011). Fetal origins of adult disease. Curr Probl Pediatr Adolesc Health Care.

[34] Henderson J, Granell R, Heron J, Sherriff A, Simpson A, Woodcock A, Strachan DP, Shaheen SO, Sterne JAC (2008). Associations of wheezing phenotypes in the first 6 years of life with atopy, lung function and airway responsiveness in mid-childhood. Thorax..

[35] Colver A, Fairhurst C, Pharoah PO (2014). Cerebral palsy. Lancet..

[36] MacLennan AH, Thompson SC, Gecz J (2015). Cerebral palsy: causes, pathways, and the role of genetic variants. Am J Obstet Gynecol.

[37] Nejat EJ, Buyuk E (2012). Reproductive technologies and the risk of birth defects. N Engl J Med.

[38] Pharoah PO, Price TS, Plomin R (2002). Cerebral palsy in twins: a national study. Arch Dis Child Fetal Neonatal Ed.

[39] Skogen JC, Overland S (2012). The fetal origins of adult disease: a narrative review of the epidemiological literature. JRSM Short Rep.

[40] Kindlund K, Thomsen SF, Stensballe LG (2010). Birth weight and risk of asthma in 3-9-year-old twins: exploring the fetal origins hypothesis. Thorax.

[41] Xu XF, Li YJ, Sheng YJ, Liu JL, Tang LF, Chen ZM (2014). Effect of low birth weight on childhood asthma: a meta-analysis. BMC Pediatr.

[42] Ullemar V, Lundholm C, Almqvist C (2015). Twins’ risk of childhood asthma mediated by gestational age and birthweight. Clin Exp Allergy.

[43] Henderson AJ (2014). Asthma and lung development: another piece in the jigsaw, but the full picture has yet to emerge. J Allergy Clin Immunol.

[44] Bisgaard H, Jensen SM, Bonnelykke K (2012). Interaction between asthma and lung function growth in early life. Am J Respir Crit Care Med.

[45] Nelson KB, Grether JK. Cerebral palsy in low-birthweight infants: etiology and strategies for prevention. Ment Retard Dev Disabil Res Rev. 1997;3(2):112–7. 10.1002/(SICI)1098-2779(1997)3:2<112::AID-MRDD2>3.0.CO;2-T.

[46] Nelson KB, Chang T (2008). Is cerebral palsy preventable?. Curr Opin Neurol.

[47] Poulsen P, Vaag AA, Kyvik KO, Moller Jensen D, Beck-Nielsen H (1997). Low birth weight is associated with NIDDM in discordant monozygotic and dizygotic twin pairs. Diabetologia..

[48] Turner S, Fielding S, Mullane D, Cox DW, Goldblatt J, Landau L, le Souef P (2014). A longitudinal study of lung function from 1 month to 18 years of age. Thorax..

[49] Graham HK, Rosenbaum P, Paneth N, Dan B, Lin JP, Damiano DL, Becher JG, Gaebler-Spira D, Colver A, Reddihough DS, Crompton KE, Lieber RL (2016). Cerebral palsy. Nat Rev Dis Primers.

[50] Pharoah PO (2001). Cerebral palsy in the surviving twin associated with infant death of the co-twin. Arch Dis Child Fetal Neonatal Ed.

